# Sequential interventions to maintain the safety and service provisions of human milk banking in India: keeping up with the call to action in response to the COVID-19 pandemic

**DOI:** 10.1186/s13006-022-00525-1

**Published:** 2022-12-14

**Authors:** Maheshwar Bhasin, Sushma Nangia, Gunjana Kumar, Abha Parihar, Srishti Goel

**Affiliations:** 1 Vatsalya Maatri Amrit Kosh, National Comprehensive Lactation Management Centre, Lady Hardinge Medical College, Connaught Circle, New Delhi, India; 2 Department of Neonatology, Lady Hardinge Medical College, Kalawati Saran Children’s Hospital, New Delhi, India

**Keywords:** Human milk bank, Breastmilk, Exclusive breastfeeding, Mothers’ own milk, Donor human milk, Lactation counselling, WhatsApp

## Abstract

**Background:**

WHO recommends donor milk as the next best choice if Mothers’ own milk (MOM) is unavailable. At our milk bank, during the COVID 19 pandemic, we observed a steep decline in the collection of donor milk, while Pasteurised Donor human milk (PDHM) demand increased. This called for active intervention.

**Methods:**

We employed the quasi-experimental quality improvement initiative. During September 2020 (baseline period) the team members identified modifiable bottlenecks and suggested interventions (using WhatsApp to increase follow up, telehealth and digital tools) which were implemented in October 2020 and the impact was evaluated till March 2021. The SMART aim was “to meet the demand (estimated as 15,000 ml/month) of donor milk for adjoining 80-bedded NICU”. Process measures were; daily amount of donor milk collected, pasteurized donor milk disbursed to NICU, number of donors and frequency of donations. The balancing measure was that the collection of donor milk should not undermine the provision of freshly expressed MOM for babies.

**Results:**

Collection of donor milk increased by 180% from baseline during the Intervention phase. This was sustained throughout the sustenance phase (November 2020 and March 2021) with an average monthly collection of 16,500 ml. Strikingly, the increased follow-up of mothers with emphasis on MOM decreased the NICU’s donor milk requirement from 13,300 ml (baseline) to 12,500 ml (intervention) to 8,300 ml (sustenance). Monitoring of daily MOM used in the NICU revealed a 32% surge from 20,000 ml (baseline) to 27,000 ml (intervention) sustained at 25,000 ml per month.

**Conclusion:**

By improving the provisions of human milk banks, near-exclusive human milk feeding can be ensured even during the pandemic time.

**Supplementary Information:**

The online version contains supplementary material available at 10.1186/s13006-022-00525-1.

## Background

Early in the pandemic, the virtual collaborative network of human milk banks and associations published a call for action to ensure safety and service provisions of human milk banking [1]. In India, services provided by human milk banks which are established as Lactation management centres [Comprehensive Lactational Management Centre (CLMC), Lactational Management Units (LMU), Lactation Support Units(LMS)] are under regulation of national guidelines [2, 3]. COVID 19 restrictions were imposed on 22 March, 2020 throughout the nation resulting in a steep decline in collection of donor human milk reported from India [3, 4].

A set of changes were introduced in the centre to curtail the effect of COVID 19 [3]. We observed a decline of 62.3% in collection of Pasteurised Donor human milk (PDHM) at our centre (44,062 ml collected between Jan to March 2020 and 16,590 ml collected between April to June 2020) while the demand rose to 137% for a quarter (34,018 ml demanded between Jan to March 2020 and 46,911 ml demanded between April to June 2020). However, even with the best efforts, the voluntary donation of human milk at our centre could only rise to 27,000 ml in next quarter i.e., July to September, which was inadequate to cater to the demand at our NICU.

Quality improvement initiatives have been proven to be effective in increasing Mothers’ Own Milk usage in level 3 Neonatal Intensive Care Units (NICU) [5] and increasing the volume of donor milk collected in an Indian human milk bank [6]. With this existing background, a sequential multidisciplinary quality improvement project was planned with the objective of improving services in three domains including removing bottlenecks in donations, increasing usage of MOM in NICU and maintaining optimum functioning of the centre.

## Methods

### Context

Our hospital is a tertiary care university hospital in India catering to 14,000 + deliveries having an 80-bedded neonatal unit in India. Vatsalya- Maatri Amrit Kosh, a state-of-the-art centre for human milk banking, was established in June 2017 and has been functioning as a CLMC following the national guidelines, Ministry of Health and Family Welfare, Government of India (MoHFW, GOI) [2].

In our centre staff comprises of 4 Senior nursing officers along with 8 nursing officers delivering lactation counselling to over 200 + lactating mother baby dyads / day at 15 different areas throughout the hospital. During COVID 19, the wards were segregated as red, orange, and green zones to separate COVID positive/ suspect and COVID negative patients. This change also posed difficulty in delivering optimum lactation counselling and consequently caused a decrease in donor human milk collected at the centre. This, in turn, diminished the ability of the centre to adequately cater to the requirement of donor human milk in the adjoining NICU.

**Fig. 1:**
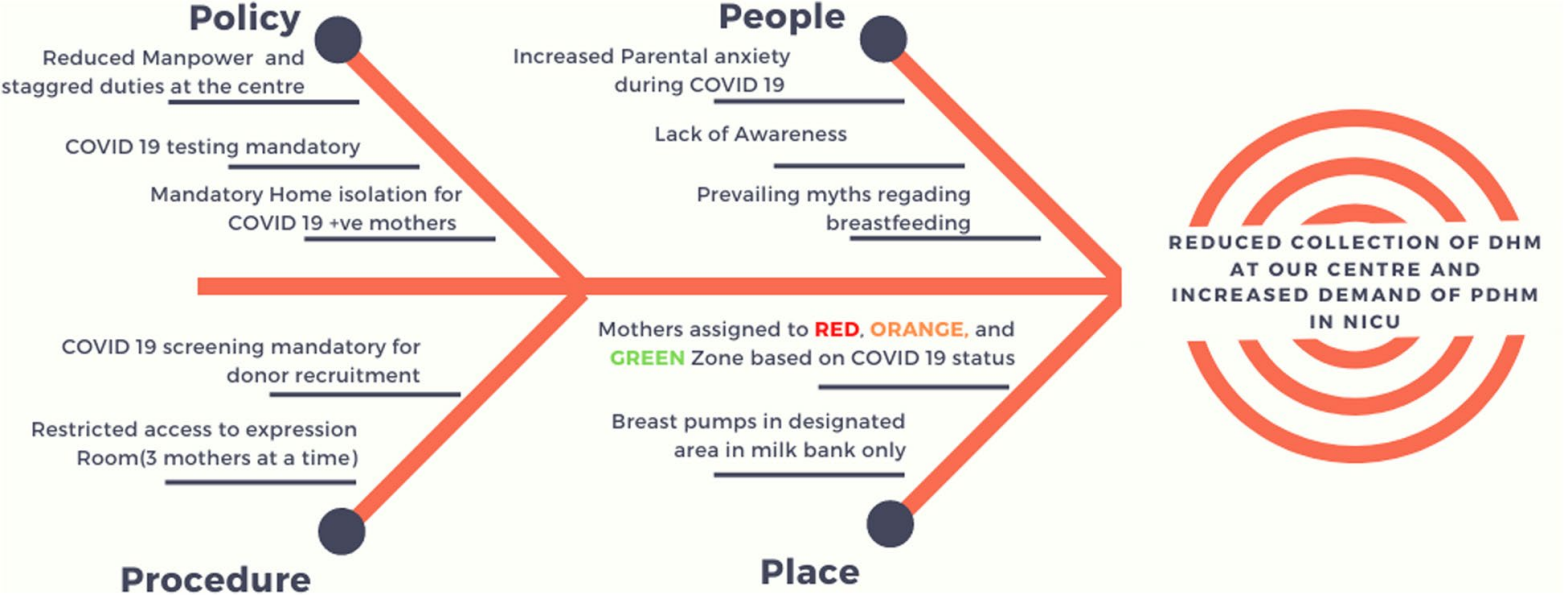
Fishbone diagram

**Fig. 2:**
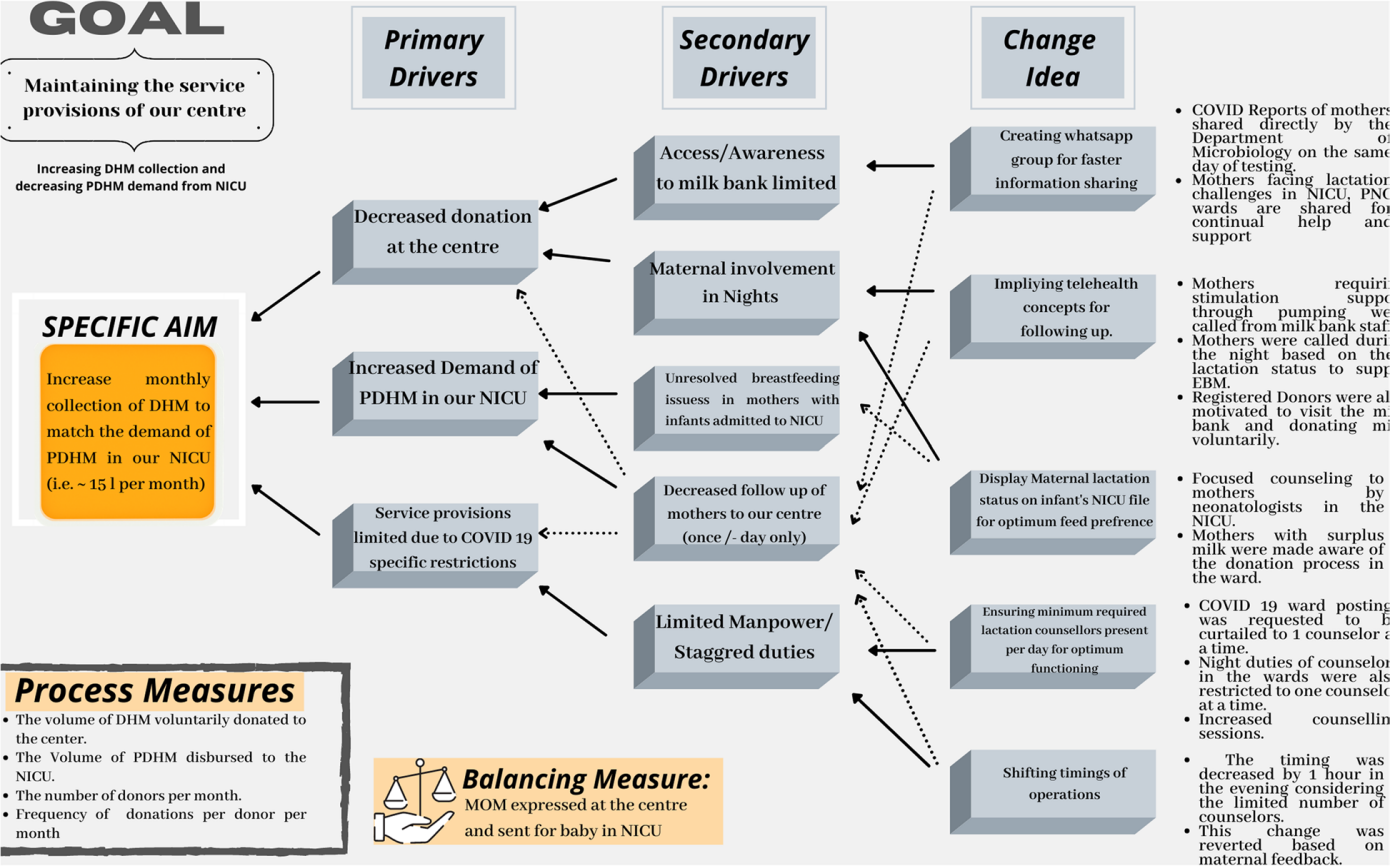
Key driver diagram

A multidisciplinary team consisting of physicians, lactation counsellors, nurses, and researchers was formed in September 2020. By retrospectively analysing the average daily demand from the NICU, the team established the SMART aim should be to cater to the demand of adjoining NICU by voluntarily collecting at least 15,000 ml of milk every month. To accomplish this goal, a model of improvement is designed for achieving an aim through learned experience and purposeful action, where teams develop a strategy of immediate and sequential changes to learn the interventions that may produce improvement [7]. A cause and effect diagram for root cause analysis using a fishbone was used for identifying the possible cause of a problem and in sorting ideas into useful categories [8]. It helped toidentify and summarize modifiable factors under 4 categories namely; People, Policy, procedure, and place **(**Fig. 1**)**. Based on this, the team created a key driver diagram to improve the overall quality and maintain the service provisions of the centre **(**Fig. 2**)**. Specific key interventions to address each key driver were developed and implemented. Baseline for the project was collected for the month of September 2020.

### Interventions

The interventions were implemented in October 2020, based on the key drivers:

- Key driver 1: Increasing donated human milk at our centre.

The focus group conducted extensive discussion to identify the bottlenecks for donations at our centre. It was repeatedly mentioned that the COVID 19 status reports took an average of 7 days. It was decided that details of mothers with negative status will be shared on a WhatsApp group. Additionally, the follow-up calls of registered donors who are still hospitalised in the maternity ward was done telephonically.

- Key driver 2: Increasing Mother’s own Milk (MOM) supply in the NICU/ Decreasing the demand of PDHM in NICU.

In addition to daily one to one counselling for mothers, the focus group identified that bottleneck was primarily night time feeding and milk availability. The NICU staff tried extensive follow up with every mother however, it was overburdening. It was suggested that lactation counsellors will share the maternal lactation status on the infant’s file as (E1 being No milk to E3 being enough milk). Initially, the mothers with E3 status were specifically counselled, called in the night and based on this, the doctors counselled the family members to ensure the provision of milk.

- Key driver 3: Optimum Functioning of the centre:

The COVID 19 pandemic led to widespread stigma associated with breastmilk and breastfeeding, which were also prevailing among lactation counsellors. We scheduled regular sessions and meetings to discuss and disseminate the latest updates in maternal and child health. To keep ourselves updated, we utilized various publicly available resources including PubMed (LitCOVID) and repositories (breastfeeding and infant feeding by John Hopkins).

In our setup, nursing officers with midwifery experience were trained to provide lactation support and work as lactation counsellors, due to the overall shortage of staff in the hospital. These nurses were frequently deputed to COVID 19 specific wards. As a result, our centre became extremely short staffed to conduct one-on-one counselling sessions for mothers. The focus group shared this concern with senior leadership of the hospital and it was decided that only one staff will be deputed for COVID 19 duties and night duty at a time.

One of the bottlenecks identified for all three key drivers was decreased communication between the milk bank and the 80 bedded unit located at two physically distinct areas as 30 and 50 bedded NICUs. Thus, three WhatsApp groups with all the staff as members were created.

### Study of Interventions

The suggested and agreed upon interventions were practised and evaluated regularly. The focus group met weekly during the baseline period (September 2020), fortnightly during the intervention period, and monthly during the sustenance phase. Daily communication was conducted using WhatsApp groups throughout the period of the study.

### Measures

The outcome measure was the total amount of milk donated to the centre, which was closely monitored and shared on daily basis. For the purview of this study, trend analysis was done utilizing donated milk calculation weekly. Another process measure considered was the analysis of the demand for PDHM received from the NICUs. The centre’s main goal is to promote exclusive breastfeeding for six months, we feared that the focus might shift towards collection of Donor human milk (DHM) and undermine the importance of freshly expressed MOM. Thus, to ensure that, a close measurement of Expressed Breast Milk (EBM) sent from the milkbank for mother’s own infant in the adjoining NICUs was noted as the balancing measure for the study.

### Analysis

The quantitative data is being presented as the means ± SD and median with 25th and 75th percentiles (interquartile range). The data normality was checked by using Kolmogorov-Smirnov test. The cases in which the data was not normal, we used non parametric tests. The comparison of the variables which were quantitative and not normally distributed in nature were analysed using Kruskal Wallis Test. For all not normally distributed data, a Post Hoc analysis by Dunn’s multiple pairwise comparison test was carried out. The data entry was done in the Microsoft EXCEL spreadsheet and the final analysis was done with the use of Statistical Package for Social Sciences (SPSS) software, IBM manufacturer, Chicago, USA, ver 21.0. For statistical significance, p value of less than 0.05 was considered as significant. Analysis was done using Microsoft Excel 2016. Statistical Process control p-charts were used to examine the process measure based on weekly data. The control limits were set according to key outcome data from September 2020 and process changes were indicated for every phase to calculate new control limit. The manuscript adheres to the applicable SQUIRE guidelines and checklist [9].

## Results

The key interventions entailed in the initiative and suggested by the focus group were implemented. At most, only one of the interventions i.e., operational timing of the centre, was required to be modified. This modification to revert to earlier timing was implemented based on the feedbacks gathered from mothers and focus group discussion.

As a result of this QI, the centre was able to collect a total of 108,426 ml donor human milk from 314 first time donors donating multiple times. Whereas, the requisition of pasteurized donor human milk from NICUs decreased considerably with only 72,990 ml of pasteurised donor human milk (PDHM) requested and disbursed. Thereby, we were able to outmatch the demand and supply of donor human milk at our centre. It was initially feared that emphasising on collection of donor human milk might lead to a reduction in expressed breast milk sent for the babies. However, through diligent efforts of the staff with an overall increase of counselling sessions and follow-up of mothers, a total of 171,239.5 ml of freshly expressed mothers’ own milk was sent for neonates admitted in NICUs during the course of the study (September 2020 to March 2021). (Table 1)

**Table 1:** Comparison of measure between baseline phase (September 2020), intervention phase (October 2020) and sustenance phase (November 2020 to March 2021)

Measure	Baseline Phase (September 2020) (*n* = 26)	Intervention Phase (October 2020) (*n* = 27)	Sustenance Phase (November 2020 to March 2021) (*n* = 128)	*P* value
**Number of counselling sessions conducted**				
Median (25th-75th percentile)	144.5(131.3–154.5)	157(150–166)	158(138.5-197.3)	0.012^a^ B vs. I:**0.024** B vs. S:**0.003** I vs. S:0.945
Range	113–176	91–198	15–269	
**Number of expressions through pumping**				
Median (25th-75th percentile)	27.5(21.3-30)	30(24.5–35.5)	30(24–37)	0.082^a^ B vs. I:0.087 B vs. S:**0.027** I vs. S:0.986
Range	13–38	15–52	8–63	
**Total number of donations**				
Median (25th-75th percentile)	3.5(2–5)	6(4-8.5)	6(5–9)	0.0002^a^ B vs. I:**0.004** B vs. S:<.**0001** I vs. S:0.654
Range	0–11	1–18	0–16	
**Volume of DHM collected at the centre (daily) (ml)**				
Median (25th-75th percentile)	377.5(160-501.3)	520(310–670)	617.5(391.3-832.8)	0.0004^a^ B vs. I:**0.02** B vs. S:<.**0001** I vs. S:0.311
Range	0-1000	60-1745	0-1705	
**Volume of PDHM disbursed to the NICU (daily) (ml)**				
Median (25th-75th percentile)	520(260–650)	390(260–650)	260(130–520)	0.004^a^ B vs. I:0.449 B vs. S:**0.003** I vs. S:**0.043**
Range	130–1040	0-910	0-1690	
**Volume of MOM sent for the baby admitted to NICU (daily) (ml)**				
Median (25th-75th percentile)	734(618-96235)	941(745-1108.25)	930(779.9–1115)	0.011^a^ B vs. I:**0.023** B vs. S:**0.003** I vs. S:0.946
Range	328–1239	507–1993	368–1910	

Test used: -^a^ = Kruskal Wallis test

**Fig. 3:**
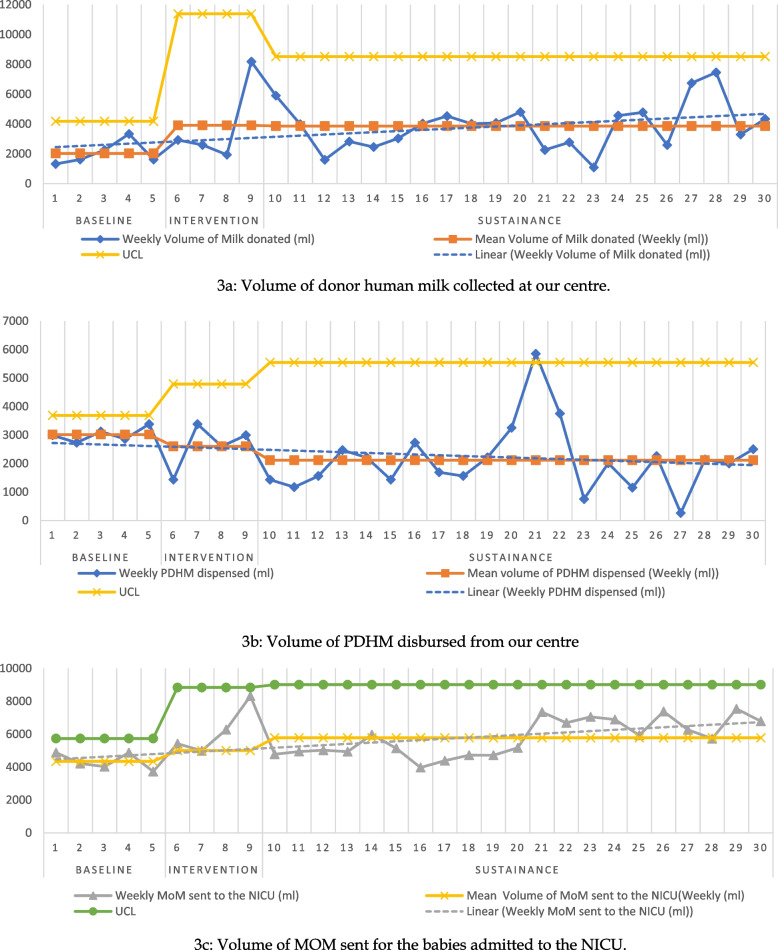
Statistical Process control charts for Process measures and balancing measure of our study. 3**a**: Volume of donor human milk collected at our centre. Figure 3**b**: Volume of PDHM disbursed from our centre. Figure 3**c**: Volume of MOM sent for the babies admitted in the NICU. (UCL: Upper control limit, LCL: Lower control limit.)

During the baseline period, every aspect was meticulously screened to identify key areas of improvement (Fig. 1). Proactive involvement of the focus group and faculty members involved in the project was the key determinant in the intervention phase. Few interventions required administrative changes and inter-departmental collaboration which took time to implement. Hence, it resulted in a sudden surge of donors and donated milk collected towards the end of the intervention phase. This surge in donor human milk (Fig. 3a) was also observed in MOM sent to the NICUs (Fig. 3c). However, once established, the interventions were monitored continuously and no modifications were required in the sustenance phase. The focus group conducted monthly meetings to reflect on the progress in the sustenance phase.

### Process measures and outcome

During the baseline phase, a total of 9,165 ml of donor human milk was collected at our centre while the total pasteurized donor human milk requested and disbursed to the adjoining NICUs was 13,130 ml (Table 1). Although, we adopted COVID specific modifications at our centre early in the pandemic [3], the situation did not improve and remained consistent with our previous observations.

Due to the initiative, in the intervention phase, we overserved a 180% increase in the amount of total donor human milk collected at our centre which amounted to 16,605 ml in October 2020 (Table 1). This increase might be the result of increased donors (33 first time donors in September 2020 to 53 first time donorsin October 2020 donating multiple times). More importantly, this increase was not translated into a decline in expressed breast milk sent for babies in NICUs. A 32% increase in EBM sent for babies in NICUs from 20,105.8 ml during the baseline phase to 26,620 ml in the intervention phase. We also observed a slim decline of 5% in PDHM requested to 12,350 ml. Thus, we were able to conservatively fulfil the demand and supply of donor human milk at our centre.

In the sustenance phase. we were able to maintain the progress in the process measures. Over the following 5 months (November 2020 to March 2021), we were able to collect a total of 82, 656 ml. We also report a remarkable decline in the PDHM requirement in the adjoining NICUs to 41,510 ml along with 124,513.7 ml of expressed breast milk sent to the babies in the two NICUs. This reflects our inclination towards the use of freshly expressed MOM. Statistical control charts for the daily volume of milk donated, volume of PDHM disbursed, and volume of freshly expressed MOM sent for babies in NICUs report a gradual increment (Fig. 3).

## Discussion

Breastfeeding and breastmilk are quintessential for infant feeding. Various studies have indicated the dampening effect of prevention strategies of COVID 19 on breastfeeding including maternal and child separation [10–12]. Oncel and colleagues concluded that parental anxiety regarding breastmilk led to early interruptions of breastfeeding practices in NICU during COVID 19 pandemic [13].

During the pandemic also WHO supported continued breastfeeding and breast milk usage and in circumstances where MOM is inadequate, WHO recommended the use of donor human milk [14]. In a quality improvement initiative, Ward and colleagues estimated that 25% of VLBW in their NICU between January 2006 to June 2010 met their feeding goals because of a copious supply of donor human milk [15].

Donor human milk is a scarce resource and should be prioritised [16, 17]. We continued operations at our centre with COVID 19 specific modifications in alignment with the guidelines [3, 18]. However, we still observed a consistent decline in collection of donor human milk at our centre. In a newspaper article in June 2020, a regional centre from Jaipur, Rajasthan also reported a significant drop in collection at their centre [3]. Similar observations of significant reduction can also be observed in recently published trend from milk bank in Vietnam [19]. In this study, we demonstrate the improvement in the functioning of the centre after the implementation of QI strategies in augmenting the use of human milk and collection of Donor human milk at a human milk bank in a tertiary healthcare centre in India. Key change ideas and interventions includes:

- *Continued mother- lactation counsellors’ interactions (Prevention should not mean separation)*
- *Telephonic follow up by nursing officers (especially during night time) and counsellors during the day.*
- *Time management by utilizing coding for quick inter-team (Neonatologists, Paediatricians, Nurses and Counsellors) updates about current lactation support needs for every mother.*
- *Inter-departmental support to ensure quick delivery of services. (using WhatsApp to update COVID negative status of mothers)*
- *Increased milk bank stakeholders’ involvement by using standard reporting formats and daily updates*

We were able to maintain a steady supply of human milk (freshly expressed MOM & PDHM) to the NICU. The quality improvement measures not only helped in increasing the donor human milk collected at the centre, it also showed a significant increment in counselling sessions conducted per day, expression of milk through electric breast pump, and also the amount of freshly expressed MOM sent for neonates admitted to the adjoining NICUs. The interventions scheduled did not involve external funding and were simple system changes, thereby, the change ideas can be sustained over a long period of time. This is reflected by a continued improvement in process measures (Fig. 3).

Counselling and encouragement by health care professionals is known to play a significant role in maintaining and sustaining breastfeeding [20, 21]. The results from our study are also consistent with other studies which found that human milk feeding was augmented by multidisciplinary staff training and their involvement [15, 22, 23]. In a landscape analysis, Sachdeva et al. highlight the wide variation in human resource available for counselling in Indian milk banks [24]. The study was done in 2016 and reported that no milk bank had > 5 lactation counsellors [24]. We are fortunate to have a team of 8 lactation counsellors, which enabled us to successfully execute this QI initiative. We utilized Johns Hopkin “COVID 19 and infant feeding” repository to stay updated and disseminate the information among our team [25]. This, along with continuous interactions helped alleviate anxiety and tackle the resistance among lactation counsellors to better equip them to counsel the mothers.

We created dedicated WhatsApp groups which significantly improved inter and intra-departmental communication. Three separate WhatsApp group were created: first one, with the microbiology department to share COVID 19 status of mothers ; second one, included the healthcare staff of the neonatology department which was used exclusively to share maternal lactation status and lactation support provided to mothers, daily PDHM requirement and feeding updates; and the third, included focus group members and milk bank staff in which daily updates and snapshot of daily numbers of mothers counselled, PDHM collected and MOM disbursed was shared with stakeholders. We observed that WhatsApp group communication improved and channelised lactation counselling efforts. Similar effect of group communications utilising WhatsApp and WeChat are reported from India [6] and China [26, 27]. We plan to continue two groups post-pandemic to sustain the progress achieved by this study.

In view of the limited staff, initially we decided to curtail the timing of centre till 5 pm. This was implemented to ensure adequate number of counsellors present daily in the milk bank; however, this change was reversed based on feedback from mothers. Unlike most high-income countries, where donors recruited at milk bank expresses their milk at home and donate repeatedly over a long period of time [28], at our centre most donors are mothers who deliver in our hospital and longitudinal donors are usually mothers whose infant is admitted to the NICU [29]. Due to COVID, we also saw a decline in number of mothers delivering in the hospital which in turn posed a challenge for donor recruitment [3]. However, the increased support and counselling to mothers in the hospital resulted in us achieving the SMART aim of this QI.

Sachdeva et al. report a median of 498,000 ml of milk annually in a public sector human milk bank in India [22]. We collected only 142,400 ml between April 2020 to March 2021 which is comparatively low compared to previous studies from 2016 [24] and 2019 [6]. However, no study from India has evaluated the impact of COVID 19 on human milk banks in India. At our centre, the majority of donation i.e., 108,400 ml (76%) was collected during the study. We are hopeful that by sustaining the change we can curtail the impact of the anticipated waves of SARS CoV 2 mutants in India [30].

The limitation of study might be that we did not directly evaluate the impact of our QI on feeding pattern in our NICUs. However, due to the lack of funding and to avoid overburdening of clinicians during COVID 19 pandemic, the data collection in NICU was not feasible and thus not included in the study. We hope to analyse the pattern retrospectively in the future. The current study was focussed on our centre, thereby, few interventions were contextually implemented.

## Conclusion

The QI initiative helped to attain the specific aim of the study. The interventions also helped the centre to maintain and safely continue the service provisions of CLMC during the pandemic. Strategically planned quality improvement initiative for local contexts can result in optimum functioning of human milk banks in challenging circumstances like COVID 19. Simple organisational change, inter- and intra-departmental collaboration and effective communication to support the lactating mothers could improve the use of human milk in NICUs. Sustainability of such interventions can be ensured by effective management of available resources.

## Supplementary Information

**Additional file 1.**

## Data Availability

All data generated or analysed during this study are included in this published article.

## Abbreviations

CLMC: Comprehensive Lactation Management Centre
COIVD 19: Coronavirus Infection disease 2019
MoHFW, GOI: Ministry of Health and Family Welfare, Government of India
MOM: Mother’s/s’ own milk
NICU: Neonatal Intensive Care Unit
PDHM: Pasteurised donor human milk
QI: Quality improvement
SQUIRE: Standards for QUality Improvement Reporting Excellence
V/LBW: Very/ low birth weight
WHO: World Health Organisation
