# Supplementary material for: Sequential interventions to maintain the safety and service provisions of human milk banking in India: keeping up with the call to action in response to the COVID-19 pandemic
Source: Int Breastfeed J. 2022 Dec 14;17:85. doi: 10.1186/s13006-022-00525-1 (PMC9748401; doi:10.1186/s13006-022-00525-1)
Supplement: Supplementary file 1 — Additional file 1. [file 13006_2022_525_MOESM1_ESM.docx]

To

Editor

International Breastfeeding Journal

Dear Madam

The authors are thankful to the reviewers and yourself for the appreciative and valuable comments.

The comments, suggestions and respective responses are tabulated below. In addition, the respective page and line number where the changes have been made are also given in the last column.

Author Team

Editor-in-Chief comments:

| 1.      Footnote- thank you for explaining what you mean by “counsellors a.k.a. nursing officers”. However, the journal style doesn’t usually include footnotes. Please explain what you mean in a proper sentence without using a footnote or “a.k.a.”. | Amended as per your suggestion. |
| --- | --- |
| 2.      “a fishbone analysis” – You’ve said “The details relevant to fish bone analysis has been included in the manuscript at page 5, line 90 to 92. The reference for the same is added”. While you have added a reference for root-cause analysis, I’ve checked this paper and fishbone analysis is just listed as ‘another method”. Please cite a primary reference about fishbone analysis here. | We apologise for the mis-referencing. The reference has been updated to appropriate study. |
| 3.      References These must be all correct before we can accept the manuscript. See the Instructions for authors and published papers. Obviously <google citations “Vancouver style”> is not working. #1. Authors’ names – as I said before <Should be six authors then “et al”>. Date of publication should not be included – just year – delete “Jul 1”. #3. International breastfeeding journal – journal title needs to be correctly capitalised. Should be 15:104. BMC journals, including IBJ, do not have issue numbers or page numbers. #4. Fix journal title and delete month. #5. Should be 4(5):e204. #6. Online ahead of print – needs doi. Please continue to check and correct every reference. Note that title of articles should be in sentence case, e.g. #20. Mothers' Decisions to Change From Formula … should be Mothers' decisions to change from formula … | Amended as per your suggestion. |
| No need to upload a copy with tracked changes. Please just submit a clean version ready for publication so we can move to accept it. | We thank you for your suggestions and support. |
